# Mild and moderate manifestations of SARS-CoV-2 infection, including hospitalization, in children and adolescents with cystic fibrosis

**DOI:** 10.31744/einstein_journal/2025AO1312

**Published:** 2025-04-25

**Authors:** Fábia Regina dos Santos, Daniela Gois Meneses, Ricardo Queiroz Gurgel, Tatiana Rodrigues de Moura, Camilla Natália Oliveira Santos, Lucas Sousa Magalhães, Alexia Ferreira Rodrigues, Maria Luiza Doria Almeida, Allan Valadão de Oliveira Britto, Angela Maria Silva

**Affiliations:** 1 Programa de Pós-Graduação em Ciências da Saúde Universidade Federal de Sergipe Aracaju SE Brazil Programa de Pós-Graduação em Ciências da Saúde, Universidade Federal de Sergipe, Aracaju, SE, Brazil.; 2 Department of Medicine Hospital Universitário Universidade Federal de Sergipe Aracaju SE Brazil Department of Medicine, Hospital Universitário, Universidade Federal de Sergipe, Aracaju, SE, Brazil.; 3 Laboratório de Imunologia e Biologia Molecular Universidade Federal de Sergipe Aracaju SE Brazil Laboratório de Imunologia e Biologia Molecular, Universidade Federal de Sergipe, Aracaju, SE, Brazil.; 4 Instituto de Ciências Biológicas e da Saúde Universidade Federal de Alagoas Maceió AL Brazil Instituto de Ciências Biológicas e da Saúde, Universidade Federal de Alagoas, Maceió, AL, Brazil.

**Keywords:** Cystic fibrosis, Virus diseases, Respiratory function tests, SARS-CoV-2, Coronavirus infections, Hospitalization, Adolescent, Adolescent, hospitalized, Child, Child, hospitalized

## Abstract

**Objective:**

This study aimed to evaluate the clinical manifestations of SARS-CoV-2 in children and adolescents with cystic fibrosis.

**Methods:**

This was a case-control analysis of clinical variables and pulmonary function test results in 43 children with cystic fibrosis, 17 (39.5%) of whom tested positive for SARS-CoV-2.

**Results:**

The infected children exhibited a higher frequency of dyspnea and cough and a greater need for hospitalization. One infected child died. Pulmonary exacerbations were more frequent among the infected children. Additional data indicated a subsequent reduction in pulmonary function in the infected children, although this was not significantly different from that in the uninfected children.

**Conclusion:**

Children with cystic fibrosis who contracted SARS-CoV-2 experienced mild to moderate symptoms and required hospitalization but generally had high recovery rates.

## In Brief

Santos et al. analyzed the clinical characteristics and pulmonary function of children with cystic fibrosis infected with SARS-CoV-2. Infected children showed higher rates of dyspnea, coughing, hospitalization, and pulmonary exacerbations. Despite a temporary decline in pulmonary function, the recovery rates matched those of the uninfected children during follow-up.

## Highlights

■ SARS-CoV-2 infection leads to mild-to-moderate disease in children with cystic fibrosis.

■ No worsening of cystic fibrosis was observed months after infection.

## Graphical abstract

**Figure:**
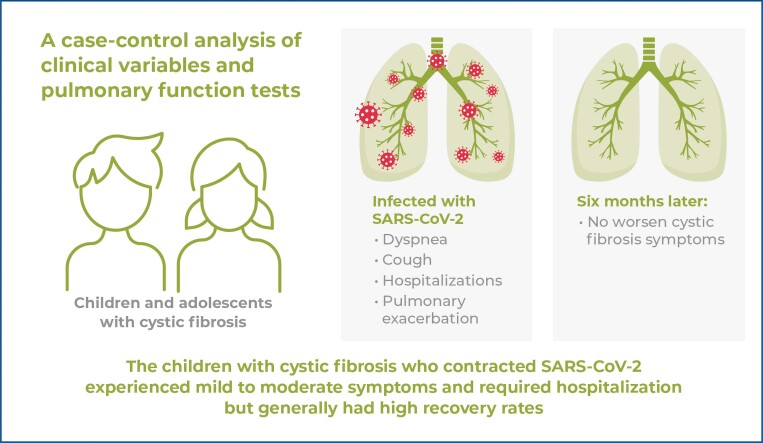

## INTRODUCTION

Cystic fibrosis (CF) is an autosomal recessive monogenic disease that affects various organs and systems of the body, particularly the gastrointestinal and pulmonary systems.^1^Pulmonary dysfunction, characterized by worsening respiratory symptoms and pulmonary exacerbations, plays a principal role in the morbidity and mortality of patients with CF, leading to reduced life expectancy.^2^Coronavirus Disease 2019 (COVID-19) is caused by Severe Acute Respiratory Syndrome Coronavirus 2 (SARS-CoV-2). Compared with other respiratory viruses, it is highly contagious and has a high fatality rate in the general population, especially among individuals with comorbidities.^3^

Previous research has demonstrated that patients with CF benefited from the social preventive measures implemented during the COVID-19 pandemic.^4^However, the long-term repercussions of SARS-CoV-2 infection in individuals with CF are still not well understood, although observational data suggest a potentially less severe course of the disease than initially assumed.^5^Thus, we hypothesized that SARS-CoV-2 infection in children with CF could lead to a worsening of respiratory function.

## OBJECTIVE

To evaluate the clinical manifestations of SARS-CoV-2 in children and adolescents with cystic fibrosis and compare their clinical characteristics before and after infection.

## METHODS

### Study design and recruitment of patients

This observational case-control study using convenience sampling involved children and adolescents attending the Cystic Fibrosis Reference Center of the *Hospital Universitário* of the *Universidade Federal de Sergipe* (CRFC/HU-EBSERH/UFS). All participants had a prior diagnosis of CF and were followed up at the reference center according to Brazilian guidelines.^6^A total of 53 children and adolescents were followed up at the reference center, representing 98% of all children with CF in the state. The study was conducted from May 2020 to May 2021, during the first wave of the COVID-19 pandemic. Nasopharyngeal swabs were collected from the participants regardless of the presence of symptoms. SARS-CoV-2 detection using RT-qPCR was performed at the Central Laboratory of Public Health.^7^Additionally, information from the periods before (6-12 months) and after (approximately 6 months) the COVID-19 episodes was retrieved from the medical records of all participants.

### Evaluation of the individuals and data collection

Data on age and sex were obtained along with clinical data, such as genotype, medications used, weight, height, body mass index (BMI), pulmonary microbiology evaluations, pulmonary exacerbations, hospitalizations, and pulmonary tests. The clinical team, which also served as the research team, collected data from routine tests conducted before and after the COVID-19 episode, as well as from additional testing during the disease period.

Pulmonary function was assessed using spirometry in patients older than 6 years in accordance with international and national recommendations.^8^During the inhalation and exhalation phases, the forced vital capacity (FVC), forced expiratory volume in the first second (FEV1), and forced expiratory flow between 25% and 75% of the FVC curve (FEF25-75%) were measured. The final mean FVC and FEV1 values were used and classified as normal, mild, moderate, moderately severe, severe, or very severe.^9^The nutritional status was also evaluated using the BMI Z-score, according to the WHO recommendations. BMI was calculated as weight divided by height squared. For all patients, the anthropometric index BMI/age (BMI/A) was calculated using Z-scores with the WHO Anthro Software version 2.0.2.

Pulmonary exacerbation was defined as the presence of at least three of the following criteria: increased coughing frequency; changes in sputum (color, volume, consistency, and hemoptysis); appearance or worsening of dyspnea; loss of appetite/weight; malaise; fatigue or lethargy; headache; pain in the sinus area; absence from school/work related to worsening of respiratory function; decreased exercise tolerance; changes in lung auscultation; changes in chest radiography results; fever; a drop in FEV1 >10% from baseline; and a drop in oxygen saturation >10% from baseline.

### Statistical analysis

The Shapiro-Wilk test was used to assess the normality of continuous variables. Fisher’s exact test or McNemar’s test was used to evaluate the independence of categorical variables. The equality of medians was tested using a *t*-test or a paired *t*-test. The equality of medians with repeated measures was tested using a two-way ANOVA. Regression methodologies were used to estimate the differences between the groups at different times. A p<0.05 was considered statistically significant for comparisons.

### Statements

#### Ethics statement

This study was performed in accordance with the Declaration of Helsinki and was approved by the Human Research Ethics Committee of the *Hospital Universitário* of the *Universidade Federal de Sergipe* (CAAE: 24372019.2.0000.5546; #3.751.710). Children and their legal guardians were invited to participate, and informed consent was obtained from all patients and their legal representatives.

## RESULTS

A total of 43 children and adolescents with CF were enrolled in the study, of whom 17 (39.5%) tested positive for SARS-CoV-2. Table 1 displays the clinical characteristics and follow-up data for both groups: those with and without COVID-19. Both groups had similar proportions of females and similar ages. Regarding the baseline clinical data, heterozygosity for the F508del allele was the most frequent (41.9%). Pancreatic insufficiency was observed in 41 participants (95.3%), and one (2.3%) presented with CF-related diabetes. Both groups had similar positive bacterial identification and isolation (p=0.206) and BMI Z-scores (p=0.207).

**Table 1:** Clinical characteristics of children and adolescents with cystic fibrosis during the COVID-19 follow-up

	CF + COVID-19 n=17 (%)	CF n=26 (%)	p value^†^
Clinical characteristics			
Sex			
Female	10 (58.8)	15 (57.7)	1.000
Male	7 (41.2)	11 (42.3)	
Age, years			
0-2 years	6 (35.3)	3 (11.5)	0.102
2-10 years	5 (29.4)	15 (57.7)	
10-18 years	6 (35.3)	8 (30.8)	
CFTR genotype			
Homozygote F508del	5 (29.4)	6 (23.1)	0.786
Heterozygote F508del	6 (35.3)	12 (46.2)	
Others	6 (35.3)	8 (30.8)	
Pancreatic insufficiency	16 (94.1)	25 (96.2)	1.000
Diabetes related to CF	0 (0)	1 (3.8)	1.000
Pulmonary bacterial isolation	9 (56.2)	19 (73.1)	0.206
BMI Z-score	-0.30 [-1.45; 0.80]	-0.08 [-0.62; 0.52]	0.207^‡^
Disease presentation and treatment			
WHO clinical scale			
Uninfected	0	26 (100)	
Ambulatory mild COVID-19	11 (64.7)	0	
Hospitalized moderated COVID-19	5 (29.4)	0	-
Hospitalized severe COVID-19	0	0	
Dead	1 (5.9)	0	
Follow-up			
Outpatient	11 (64.7)	26 (100)	0.002
Hospitalization	6 (35.3)	0 (0)	0.002
Symptomatology	11 (64.7)	4 (15.4)	0.002
Fever	3 (17.6)	0 (0)	0.055
Dyspnea	4 (23.5)	0 (0)	0.019
Cough	8 (47.1)	3 (11.5)	0.014
Anorexia	3 (17.6)	0 (0)	0.055
Weight loss	1 (5.9)	0 (0)	0.395
Malaise, fatigue, or lethargy	1 (5.9)	0 (0)	0.395
Cephalgia	2 (11.8)	1 (3.8)	0.552
Pharyngitis	1 (5.9)	0 (0)	0.395
Gastrointestinal symptoms	1 (5.9)	0 (0)	0.395
Pulmonary exacerbation	5 (29.4)	1 (3.8)	0.028
Use of antibiotics	4 (23.5)	7 (28)	1.000
Corticotherapy			
Inhaled	0	7 (26.9)	0.031
Systemic	0	1 (4)	1.000
Antiviral treatment	0	0	-
Outcome			
Recovery	16 (94.1)	26 (100)	0.395
Death	1 (5.9)	0 (0)	

^†^ Fisher’s exact test; ^‡^ Paired *t*-test. The continuous data are presented as medians (interquartile ranges [25-75%]).

BMI: body mass index; CF: cystic fibrosis.

Among the infected patients, 11 (64.7%) were monitored at home and six (35.3%) required hospitalization. None of the uninfected children required hospitalization during the same time. Among the hospitalized patients with COVID-19, three (17.6%) required supplemental oxygen therapy, and one (5.9) required ICU care. Sixteen (94.1%) patients with COVID-19 recovered, and one (5.9%) died. None of the children without SARS-CoV-2 infection were hospitalized or died. There was a significant difference between the infected and uninfected patients in terms of the occurrence of pulmonary exacerbation (p=0.028) and symptomatology (p=0.002), with the appearance or worsening of dyspnea (p=0.019) and appearance or worsening of cough (p=0.014) being the most common symptoms. The use of inhaled corticosteroids was significantly higher in the uninfected group (p=0.031).

**Figure 1:**
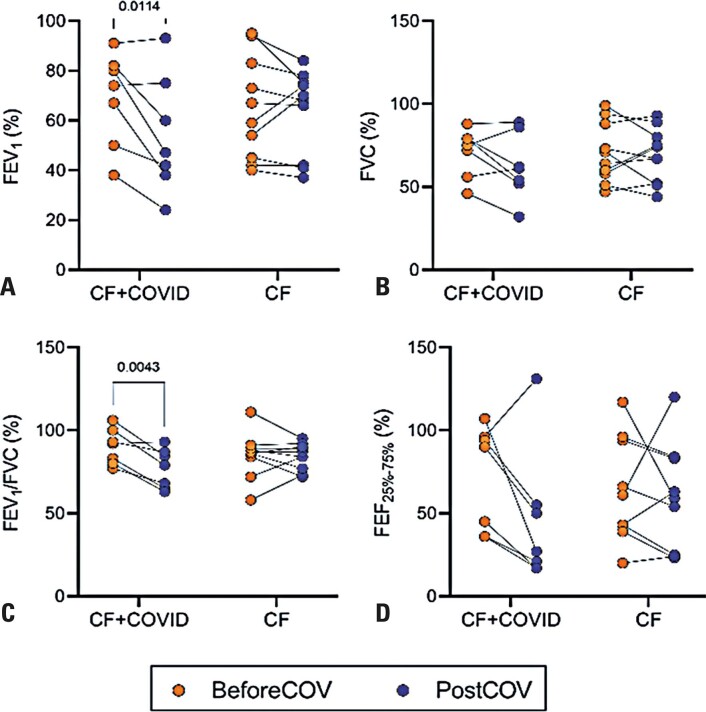
Pulmonary function test in children and adolescents with cystic fibrosis before and after the COVID-19 episodes. Forced expiratory volume in the first second (FEV1) (A), forced vital capacity (FVC) (B), the ratio between FEV1 and FVC (C), and forced expiratory flow between 25% and 75% of the FVC curve (FEF25-75%) (D). The data were retrieved from the medical records. The tests were performed for children and adolescents who met the criteria for examination. Each dot represents one included individual. The orange color indicates data from before the COVID-19 episode, and the blue color indicates data from after the COVID-19 episode. Two-way ANOVA was performed, including comparisons between groups and over time

Complementarily, data were recovered from the periods before and after the COVID-19 episode in both groups. Thus, the BMI Z-score, level of positive bacterial isolation, and level of pulmonary exacerbation were similar in both groups when comparing the periods before and after the infection (Table 2). There was an increase in the BMI Z-score in the CF Group when comparing the periods before and after the intervention (p=0.044). In addition, pulmonary function parameters were evaluated in individuals who met the spirometry criteria (Figure 1). Patients with SARS-CoV-2 infection showed lower levels of FEV1 and FEV1/FVC ratio in the period after the COVID-19 episode than in the period before (p=0.011 and p=0.004, Figure 1A and 1C, respectively). There were no alterations in the other parameters, FVC and FEF25-75%, in the infected group and no alterations in the CF Group. Moreover, no differences were observed between the groups during either evaluation period.

**Table 2:** Clinical parameters of cystic fibrosis recorded before and after the COVID-19 episode in children and adolescents included in the study

Variables	CF + COVID-19			CF			p value
	Before	After	p value	Before	After	p value	
BMI Z-score	-0.40 [-1.42; 0.33]	-0.31 [-1.60; 0.75]	>0.999^†^	-0.50 [-1.60; 0.38]	0.00 [-0.77; 0.46]	0.044^†^	0.199^†^
Pulmonary bacterial isolation*	9 (52.9)	7 (41.2)	0.479^‡^	14 (63.6)	18 (81.1)	0.763^‡^	0.285^‡^
Pulmonary exacerbation	1 (5.9)	5 (29.4)	0.200^£^	2 (7.7)	5 (19.2)	0.400^£^	0.562^£^

*Some individuals did not have microbiology test results in their records during the study period; ^†^ Two-way ANOVA with repeated measures with Bonferroni’s multiple comparison test; ^‡^ Fisher’s exact test for within-group associations and logistic regression with interaction terms for comparison between groups over time; ^£^ McNemar’s test for paired associations and generalized estimating equations for comparisons between groups over time.

Continuous data, median [interquartile range (25 - 75%)]; Categorical data, number of individuals (%).

BMI: body mass index; CF: cystic fibrosis.

## DISCUSSION

Cystic fibrosis is a chronic disease that primarily affects the lungs, making respiratory infectious diseases a significant risk factor for patients with CF.^10^In this study, children and adolescents infected with SARS-CoV-2 exhibited a mild clinical course of COVID-19 as indicated by the low ICU admission rate and moderate symptomatology, primarily presenting with cough and dyspnea. Similar and contrasting results have been observed in other healthcare settings across various countries.^5,11-14^

SARS-CoV-2 generally causes asymptomatic or mild symptoms and fewer deaths in children and adolescents compared to adults. Factors such as a lower binding capacity to the ACE2 receptor in children, enhanced activation and training of innate immunity due to frequent viral infections and vaccinations, and the absence of endothelial damage in healthy children might contribute to a more controlled disease course.^15^ Additionally, thick respiratory secretions, the respiratory microbiota, and viral-bacterial interactions in the lungs may serve as protective factors in patients with CF.^16^

In this study, children with CF who developed COVID-19 showed worsened symptoms compared to their pre-infection status, unlike non-infected children with CF. Further analysis revealed that despite an increased need for hospitalization during the acute phase, these patients generally experienced favorable clinical outcomes, with high recovery rates and low mortality. Additionally, a significant increase in pulmonary exacerbation was observed among virus-infected children, particularly those who were hospitalized. These results, along with reductions in the FEV1 and FEV1/FVC ratio, indicate a subsequent impairment in pulmonary function in these infected children. However, it is important to note that the data analyzed were obtained from the months before and after the COVID-19 episode, which could be attributed to other conditions in children with CF, who typically show lower levels on spirometry tests than other children.^17^ Moreover, children with COVID-19 and CF had similar BMI Z-scores before and after infection, whereas those with CF who were not infected exhibited an increase in the score, suggesting progression in development and nutritional status. The BMI Z-score and nutritional assessment in children with CF are important for evaluating clinical development in this group.^18^ However, although the longitudinal analysis indicated an impairment in BMI and some pulmonary parameters in children infected with SARS-CoV-2, we cannot attribute these changes solely to the infection, as there were no significant differences between the groups.

It is important to note that the patients with CF received adequate care during social isolation. Preventive measures were implemented by reference centers following national and international guidelines, including the adoption of interdisciplinary telemedicine, which likely helped maintain patient stability.^4,13^Additionally, reduced exposure to other viruses due to decreased social interactions and the increased presence of caregivers at home during the isolation period may have further contributed to the stable condition of the patients.

Despite the positive outcomes reported, this study had several limitations. The analysis at a limited time point may not have fully captured the long-term impact of the SARS-CoV-2 infection in this patient cohort. Additionally, a more comprehensive evaluation involving a larger number of patients throughout the study may have reduced data variability, given the small sample size of this study. Moreover, further evaluation of pulmonary function and additional SARS-CoV-2 testing may have enhanced the data.

## CONCLUSION

In summary, children with cystic fibrosis who were infected with SARS-CoV-2 experienced mild-to-moderate symptoms, such as increased dyspnea and cough frequency; however, they demonstrated a high recovery rate.

## References

[1] Almeida Matos B, Martins RC (2019). Cystic Fibrisis: a Literature Review. Braz J Sur Clin Res.

[2] Ramos KJ, Smith PJ, McKone EF, Pilewski JM, Lucy A, Hempstead SE, Tallarico E, Faro A, Rosenbluth DB, Gray AL, Dunitz JM, CF Lung Transplant Referral Guidelines Committee (2019). Lung transplant referral for individuals with cystic fibrosis: Cystic Fibrosis Foundation consensus guidelines. J Cyst Fibros.

[3] Stanton BA, Hampton TH, Ashare A (2020). SARS-CoV-2 (COVID-19) and cystic fibrosis. Am J Physiol Lung Cell Mol Physiol.

[4] Colombo C, Burgel PR, Gartner S, van Koningsbruggen-Rietschel S, Naehrlich L, Sermet-Gaudelus I (2020). Impact of COVID-19 on people with cystic fibrosis. Lancet Respir Med.

[5] Bain R, Cosgriff R, Zampoli M, Elbert A, Burgel PR, Carr SB (2021). Clinical characteristics of SARS-CoV-2 infection in children with cystic fibrosis: an international observational study. J Cyst Fibros.

[6] Athanazio RA, Silva LV, Vergara AA, Ribeiro AF, Riedi CA, Procianoy ED, Adde FV, Reis FJ, Ribeiro JD, Torres LA, Fuccio MB, Epifanio M, Firmida MC, Damaceno N, Ludwig-Neto N, Maróstica PJ, Rached SZ, Melo SF, Grupo de Trabalho das Diretrizes Brasileiras de Diagnóstico e Tratamento da Fibrose Cística (2017). Brazilian guidelines for the diagnosis and treatment of cystic fibrosis. J Bras Pneumol.

[7] Santos CN, Caldas GC, de Oliveira FA, da Silva AM, da Silva JS, da Silva RL (2023). COVID-19 recurrence is related to disease-early profile T cells while detection of anti-S1 IgG is related to multifunctional T cells. Med Microbiol Immunol.

[8] Diretrizes para Testes de Função Pulmonar (2002). J Bras Pneumol.

[9] Pellegrino R, Viegi G, Brusasco V, Crapo RO, Burgos F, Casaburi R (2005). Interpretative strategies for lung function tests. Eur Respir J.

[10] de Souza TH, Nadal JA, Nogueira RJ, Pereira RM, Brandão MB (2020). Clinical manifestations of children with COVID-19: a systematic review. Pediatr Pulmonol.

[11] Mondejar-Lopez P, Quintana-Gallego E, Giron-Moreno RM, Cortell-Aznar I, Ruiz de Valbuena-Maiz M, Diab-Caceres L, Prados-Sanchez C, Alvarez-Fernandez A, Garcia-Marcos PW, Peñalver-Mellado C, Pastor-Vivero MD, Olveira C, Lopez-Neyra A, Castillo-Corullon S, Palma-Milla S, Perez-Ruiz E, Sole-Jover A, Barrio MI, Sanchez-Solis M, Asensio de la Cruz Ó, CF-COVID19-Spain Registry Group (2020). Impact of SARS-CoV-2 infection in patients with cystic fibrosis in Spain: Incidence and results of the national CF-COVID19-Spain survey. Respir Med.

[12] Páez-Velásquez JS, Romero-Uribe IE, Castilla-Peón MF, Lezana-Fernández JL, Chávez-López A (2021). SARS-CoV-2 infection in a pediatric patient with cystic fibrosis. Bol Med Hosp Infant Mex.

[13] Hamad SG, Kammouh H, Alamri M, Zahraldin K (2023). The clinical features and impact of SARS-CoV-2/COVID-19 infection in children with Cystic Fibrosis (CF): A Qatari experience. Qatar Med J.

[14] Doumit M, Chuang S, Middleton P, Selvadurai H, Sivam S, Ruseckaite R (2023). Clinical outcomes of adults and children with cystic fibrosis during the COVID-19 pandemic. J Cyst Fibros.

[15] Dioguardi M, Cazzolla AP, Arena C, Sovereto D, Caloro GA, Dioguardi A (2021). Innate Immunity in Children and the Role of ACE2 Expression in SARS-CoV-2 Infection. Pediatr Rep.

[16] Manti S, Parisi GF, Papale M, Mulè E, Aloisio D, Rotolo N (2021). Looking beyond pulmonary disease in COVID-19: A lesson from patients with cystic fibrosis. Med Hypotheses.

[17] Ducati GC, Cardoso J, Ferrazeane EP, Schivinski CI (2024). Respiratory system parameters in children with low severity cystic fibrosis: is there early involvement in relation to healthy peers?. Rev Paul Pediatr.

[18] Neri LCL, Simon MI, Ambrósio VL, Barbosa E, Garcia MF, Mauri JF, Guirau RR, Neves MA, Cunha CA, Nogueira MC, Alves AC, Gurmini J, Servidoni MF, Epifanio M, Athanazio R, Grupo de Trabalho das Diretrizes Brasileiras de Nutrição em Fibrose Cística (2022). Brazilian Guidelines for Nutrition in Cystic Fibrosis. einstein (Sao Paulo).

